# SCY-247, a novel second-generation triterpenoid antifungal, demonstrates high *in vitro* activity against genetically diverse *Candida auris* isolates, including *FKS1* mutants

**DOI:** 10.1093/jac/dkaf240

**Published:** 2025-07-17

**Authors:** Bram Spruijtenburg, Theun de Groot, Eelco F J Meijer

**Affiliations:** Department of Medical Microbiology, Radboudumc, Nijmegen, The Netherlands; Radboudumc-CWZ Center of Expertise for Mycology, Nijmegen, The Netherlands; Department of Medical Microbiology and Immunology, Canisius-Wilhelmina Hospital (CWZ)/Dicoon, Nijmegen, The Netherlands; Radboudumc-CWZ Center of Expertise for Mycology, Nijmegen, The Netherlands; Department of Medical Microbiology and Immunology, Canisius-Wilhelmina Hospital (CWZ)/Dicoon, Nijmegen, The Netherlands; Department of Medical Microbiology, Radboudumc, Nijmegen, The Netherlands; Radboudumc-CWZ Center of Expertise for Mycology, Nijmegen, The Netherlands; Department of Medical Microbiology and Immunology, Canisius-Wilhelmina Hospital (CWZ)/Dicoon, Nijmegen, The Netherlands

*Candida auris* (also known as *Candidozyma auris*) causes large and persistent outbreaks in healthcare settings and is associated with a high mortality rate in patients with invasive infections. Six genetically distinct clades have been reported that differ in their genetic background and vary in their virulence, antifungal resistance and capacity to cause an outbreak.^1,2^ Treatment options are limited owing to few approved antifungals, especially as resistance development during treatment has been reported for multiple drug classes, including echinocandins, the first-line therapy for systemic *C. auris* infections^1^. With resistance rates steadily increasing, there is an urgent need to identify novel drugs for future patient care.

Triterpenoids comprise a new antifungal class that inhibit 1,3-β-glucan biosynthesis, a similar target to that of echinocandins.^3^ However, binding site profiles are different between the classes and while echinocandins are given IV, triterpenoids can be taken orally and demonstrate better tissue distribution. The first-in-class triterpenoid, ibrexafungerp (former SCY-078), was shown to inhibit the growth of different yeasts including several echinocandin-resistant isolates. Currently, a second-generation triterpenoid SCY-247 is under development with further enhanced tissue penetration. Ibrexafungerp and SCY-247 have demonstrated potent *in vitro* activity against a limited number of *C. auris* isolates, albeit with unknown information on genetic background or mutations in *FKS1*, encoding 1,3-β-glucan synthase.^4^ Therefore, we evaluated the *in vitro* activity of the second-generation triterpenoid SCY-247 against a genetically diverse collection of *C. auris* isolates, including *FKS1* mutants.

Sixty-five *C. auris* isolates from 14 countries, spanning clades I to V as determined with short tandem repeat (STR) genotyping, were included. *In vitro* antifungal susceptibility testing (AFST) against nine antifungals was conducted according to EUCAST E.Def v7.4 guidelines. SCY-247 powder was dissolved in DMSO (VWR, Amsterdam, The Netherlands) to a stock concentration of 3200 mg/L. Tested drug concentrations ranged from 0.002 to 16 mg/L. MICs were read with a spectrometer at 530 nm after 24 h of incubation. *FKS1* hotspots 1, 2 and 3 were analysed for mutations using WGS or Sanger sequencing as previously described.^5,6^ Genomic data were made available under NCBI Genbank accession numbers PQ882627–PQ882695 and SRA PRJNA982799.

The 65 *C. auris* isolates from clades I–V demonstrated highly variable *in vitro* MICs of azoles with following ranges (in mg/L): 2 to  ≥ 64 (fluconazole), 0.008–8 (voriconazole), 0.008–0.25 (itraconazole), 0.008–0.25 (posaconazole) and 0.008–1 (isavuconazole). The amphotericin B range was narrower, with MICs of 0.25–1 mg/L (Table 1, available as Supplementary data at *JAC* Online). MICs of micafungin and anidulafungin varied highly, with ranges of 0.016 to  ≥ 8 mg/L and 0.016 to  ≥ 8 mg/L, respectively (Figure 1a). The *FKS1* mutations S639T/Y/P/F, M690 V and Δ635F coincided with elevated MICs (MIC_50_ of 8 mg/L for both drugs) compared with WT isolates (MIC_50_ of 0.03 mg/L for both drugs). SCY-247 also demonstrated robust *in vitro* activity against all five clades, with a MIC range of 0.031–4 mg/L, showing an MIC_50_ of 0.125 mg/L for the WT isolates and 1 mg/L for the *FKS1* mutants (Figure 1b). The 8-fold difference of MIC_50_ between WT and mutant isolates for SCY-247 was lower than the 256-fold difference observed with both micafungin and anidulafungin. Comparing individual *FKS1* mutants, we found that for *FKS1^Δ635F^* the SCY-247 MIC was 4 mg/L and for both echinocandins the MIC was ≥8 mg/L. Except for a single S639Y isolate, which showed low MICs (0.25 mg/L) of all drugs, the other mutant isolates (with *FKS1^S639T/Y/F/P^* and *FKS1^M690V^*) demonstrated lower MICs of SCY-247 (0.25–2 mg/L) compared with the echinocandins (2 to  ≥ 8 mg/L).

**Figure 1.**
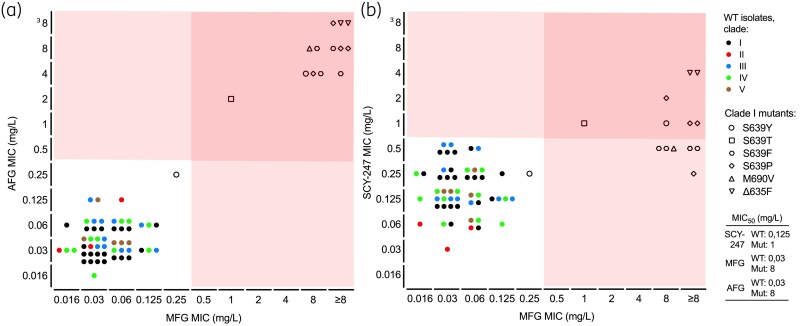
*In vitro* antifungal susceptibility testing of *Candida auris* against echinocandins and SCY-247. MICs against echinocandins and SCY-247 were determined for all *C. auris* isolates (*n*=65) according to EUCAST E.Def v7.4 guidelines and plotted in mg/L between echinocandins (a) and the echinocandin micafungin and SCY-247 (b). Different dot colours refer to the different *C. auris* clades, symbols to different *FKS1* mutations and red background to non-WT MIC values. MIC_50_, MIC at which growth was inhibited in 50% of isolates; AFG, anidulafungin; MFG, micafungin; WT, *FKS1* wild-type isolates; Mut, *FKS1* mutant isolates.

We demonstrated that the SCY-247 MIC_50_ differed 8-fold between WT and *FKS1* mutant isolates, while this was 256-fold for the echinocandins. This indicates that these *FKS1* mutations strongly reduce the susceptibility of *C. auris* to echinocandins, as is also shown *in vivo*, but have a modest impact on the efficacy of SCY-247, at least *in vitro*.^6^ This is likely due to SCY-247’s different binding site profile. While the SCY-247 MICs for virtually all *FKS1* mutants were lower compared with echinocandins, this higher *in vitro* activity does not necessarily reflect higher efficacy *in vivo*, although they are from the same pharmacological class.^7^ Furthermore, *in vitro* SCY-247 showed potent activity across all tested clades with WT *FKS1*, while azole antifungal resistance rates highly varied between isolates. Also, echinocandins only reach the urinary tract in subtherapeutic concentrations, which, as a consequence, is known as a sanctuary site facilitating resistance development for this class.^5^ Importantly, SCY-247 was designed to achieve higher tissue and potentially urinary concentrations (to be confirmed in clinical investigations), aiming to introduce a novel systemic treatment option with lower potential for resistance development.^4^ Thus, with a Phase I trial currently underway, this new antifungal could be a promising option to treat patients infected by susceptible and even echinocandin-resistant *C. auris* isolates. Nonetheless, pre-clinical *in vivo* studies are first needed to explore potential clinical usage. In addition, the efficacy against clade VI isolates and the effect of *FKS1* mutations on SCY-247 in isolates other than clade I also remains to be assessed. Altogether, the novel antifungal SCY-247 demonstrated potent *in vitro* activity against genetically diverse *C. auris* isolates, including several *FKS1* mutants with elevated echinocandin MICs.

## Supplementary Material

dkaf240_Supplementary_Data

## Supplementary data

Table S1 and Figure S1 are available as Supplementary data at *JAC*  Online.
