# Supplementary material for: SCY-247, a novel second-generation triterpenoid antifungal, demonstrates high in vitro activity against genetically diverse Candida auris isolates, including FKS1 mutants
Source: J Antimicrob Chemother. 2025 Jul 17;80(11):3165–6. doi: 10.1093/jac/dkaf240 (PMC12598769; doi:10.1093/jac/dkaf240)
Supplement: dkaf240_Supplementary_Data [file dkaf240_supplementary_data.zip › Figure S1.pptx]

## Slide 1
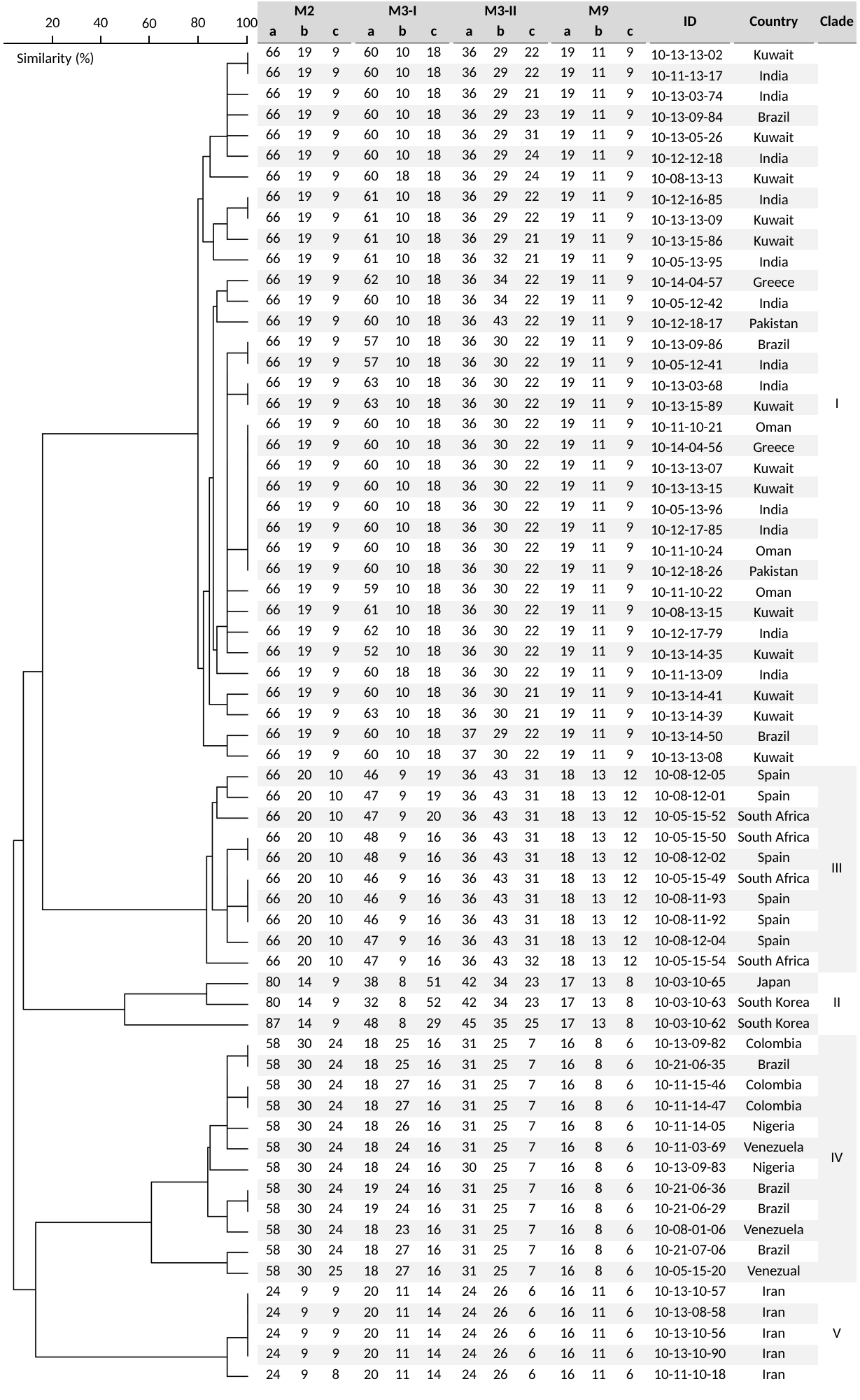

| M2 | | | | M3-I | | | | M3-II | | | | M9 | | | | ID | | Country | | Clade |
| --- | --- | --- | --- | --- | --- | --- | --- | --- | --- | --- | --- | --- | --- | --- | --- | --- | --- | --- | --- | --- |
| a | b | c | | a | b | c | | a | b | c | | a | b | c | | | | | | |
| 66 | 19 | 9 | | 60 | 10 | 18 | | 36 | 29 | 22 | | 19 | 11 | 9 | | 10-13-13-02 | | Kuwait | | I |
| 66 | 19 | 9 | | 60 | 10 | 18 | | 36 | 29 | 22 | | 19 | 11 | 9 | | 10-11-13-17 | | India | | |
| 66 | 19 | 9 | | 60 | 10 | 18 | | 36 | 29 | 21 | | 19 | 11 | 9 | | 10-13-03-74 | | India | | |
| 66 | 19 | 9 | | 60 | 10 | 18 | | 36 | 29 | 23 | | 19 | 11 | 9 | | 10-13-09-84 | | Brazil | | |
| 66 | 19 | 9 | | 60 | 10 | 18 | | 36 | 29 | 31 | | 19 | 11 | 9 | | 10-13-05-26 | | Kuwait | | |
| 66 | 19 | 9 | | 60 | 10 | 18 | | 36 | 29 | 24 | | 19 | 11 | 9 | | 10-12-12-18 | | India | | |
| 66 | 19 | 9 | | 60 | 18 | 18 | | 36 | 29 | 24 | | 19 | 11 | 9 | | 10-08-13-13 | | Kuwait | | |
| 66 | 19 | 9 | | 61 | 10 | 18 | | 36 | 29 | 22 | | 19 | 11 | 9 | | 10-12-16-85 | | India | | |
| 66 | 19 | 9 | | 61 | 10 | 18 | | 36 | 29 | 22 | | 19 | 11 | 9 | | 10-13-13-09 | | Kuwait | | |
| 66 | 19 | 9 | | 61 | 10 | 18 | | 36 | 29 | 21 | | 19 | 11 | 9 | | 10-13-15-86 | | Kuwait | | |
| 66 | 19 | 9 | | 61 | 10 | 18 | | 36 | 32 | 21 | | 19 | 11 | 9 | | 10-05-13-95 | | India | | |
| 66 | 19 | 9 | | 62 | 10 | 18 | | 36 | 34 | 22 | | 19 | 11 | 9 | | 10-14-04-57 | | Greece | | |
| 66 | 19 | 9 | | 60 | 10 | 18 | | 36 | 34 | 22 | | 19 | 11 | 9 | | 10-05-12-42 | | India | | |
| 66 | 19 | 9 | | 60 | 10 | 18 | | 36 | 43 | 22 | | 19 | 11 | 9 | | 10-12-18-17 | | Pakistan | | |
| 66 | 19 | 9 | | 57 | 10 | 18 | | 36 | 30 | 22 | | 19 | 11 | 9 | | 10-13-09-86 | | Brazil | | |
| 66 | 19 | 9 | | 57 | 10 | 18 | | 36 | 30 | 22 | | 19 | 11 | 9 | | 10-05-12-41 | | India | | |
| 66 | 19 | 9 | | 63 | 10 | 18 | | 36 | 30 | 22 | | 19 | 11 | 9 | | 10-13-03-68 | | India | | |
| 66 | 19 | 9 | | 63 | 10 | 18 | | 36 | 30 | 22 | | 19 | 11 | 9 | | 10-13-15-89 | | Kuwait | | |
| 66 | 19 | 9 | | 60 | 10 | 18 | | 36 | 30 | 22 | | 19 | 11 | 9 | | 10-11-10-21 | | Oman | | |
| 66 | 19 | 9 | | 60 | 10 | 18 | | 36 | 30 | 22 | | 19 | 11 | 9 | | 10-14-04-56 | | Greece | | |
| 66 | 19 | 9 | | 60 | 10 | 18 | | 36 | 30 | 22 | | 19 | 11 | 9 | | 10-13-13-07 | | Kuwait | | |
| 66 | 19 | 9 | | 60 | 10 | 18 | | 36 | 30 | 22 | | 19 | 11 | 9 | | 10-13-13-15 | | Kuwait | | |
| 66 | 19 | 9 | | 60 | 10 | 18 | | 36 | 30 | 22 | | 19 | 11 | 9 | | 10-05-13-96 | | India | | |
| 66 | 19 | 9 | | 60 | 10 | 18 | | 36 | 30 | 22 | | 19 | 11 | 9 | | 10-12-17-85 | | India | | |
| 66 | 19 | 9 | | 60 | 10 | 18 | | 36 | 30 | 22 | | 19 | 11 | 9 | | 10-11-10-24 | | Oman | | |
| 66 | 19 | 9 | | 60 | 10 | 18 | | 36 | 30 | 22 | | 19 | 11 | 9 | | 10-12-18-26 | | Pakistan | | |
| 66 | 19 | 9 | | 59 | 10 | 18 | | 36 | 30 | 22 | | 19 | 11 | 9 | | 10-11-10-22 | | Oman | | |
| 66 | 19 | 9 | | 61 | 10 | 18 | | 36 | 30 | 22 | | 19 | 11 | 9 | | 10-08-13-15 | | Kuwait | | |
| 66 | 19 | 9 | | 62 | 10 | 18 | | 36 | 30 | 22 | | 19 | 11 | 9 | | 10-12-17-79 | | India | | |
| 66 | 19 | 9 | | 52 | 10 | 18 | | 36 | 30 | 22 | | 19 | 11 | 9 | | 10-13-14-35 | | Kuwait | | |
| 66 | 19 | 9 | | 60 | 18 | 18 | | 36 | 30 | 22 | | 19 | 11 | 9 | | 10-11-13-09 | | India | | |
| 66 | 19 | 9 | | 60 | 10 | 18 | | 36 | 30 | 21 | | 19 | 11 | 9 | | 10-13-14-41 | | Kuwait | | |
| 66 | 19 | 9 | | 63 | 10 | 18 | | 36 | 30 | 21 | | 19 | 11 | 9 | | 10-13-14-39 | | Kuwait | | |
| 66 | 19 | 9 | | 60 | 10 | 18 | | 37 | 29 | 22 | | 19 | 11 | 9 | | 10-13-14-50 | | Brazil | | |
| 66 | 19 | 9 | | 60 | 10 | 18 | | 37 | 30 | 22 | | 19 | 11 | 9 | | 10-13-13-08 | | Kuwait | | |
| 66 | 20 | 10 | | 46 | 9 | 19 | | 36 | 43 | 31 | | 18 | 13 | 12 | | 10-08-12-05 | | Spain | | III |
| 66 | 20 | 10 | | 47 | 9 | 19 | | 36 | 43 | 31 | | 18 | 13 | 12 | | 10-08-12-01 | | Spain | | |
| 66 | 20 | 10 | | 47 | 9 | 20 | | 36 | 43 | 31 | | 18 | 13 | 12 | | 10-05-15-52 | | South Africa | | |
| 66 | 20 | 10 | | 48 | 9 | 16 | | 36 | 43 | 31 | | 18 | 13 | 12 | | 10-05-15-50 | | South Africa | | |
| 66 | 20 | 10 | | 48 | 9 | 16 | | 36 | 43 | 31 | | 18 | 13 | 12 | | 10-08-12-02 | | Spain | | |
| 66 | 20 | 10 | | 46 | 9 | 16 | | 36 | 43 | 31 | | 18 | 13 | 12 | | 10-05-15-49 | | South Africa | | |
| 66 | 20 | 10 | | 46 | 9 | 16 | | 36 | 43 | 31 | | 18 | 13 | 12 | | 10-08-11-93 | | Spain | | |
| 66 | 20 | 10 | | 46 | 9 | 16 | | 36 | 43 | 31 | | 18 | 13 | 12 | | 10-08-11-92 | | Spain | | |
| 66 | 20 | 10 | | 47 | 9 | 16 | | 36 | 43 | 31 | | 18 | 13 | 12 | | 10-08-12-04 | | Spain | | |
| 66 | 20 | 10 | | 47 | 9 | 16 | | 36 | 43 | 32 | | 18 | 13 | 12 | | 10-05-15-54 | | South Africa | | |
| 80 | 14 | 9 | | 38 | 8 | 51 | | 42 | 34 | 23 | | 17 | 13 | 8 | | 10-03-10-65 | | Japan | | II |
| 80 | 14 | 9 | | 32 | 8 | 52 | | 42 | 34 | 23 | | 17 | 13 | 8 | | 10-03-10-63 | | South Korea | | |
| 87 | 14 | 9 | | 48 | 8 | 29 | | 45 | 35 | 25 | | 17 | 13 | 8 | | 10-03-10-62 | | South Korea | | |
| 58 | 30 | 24 | | 18 | 25 | 16 | | 31 | 25 | 7 | | 16 | 8 | 6 | | 10-13-09-82 | | Colombia | | IV |
| 58 | 30 | 24 | | 18 | 25 | 16 | | 31 | 25 | 7 | | 16 | 8 | 6 | | 10-21-06-35 | | Brazil | | |
| 58 | 30 | 24 | | 18 | 27 | 16 | | 31 | 25 | 7 | | 16 | 8 | 6 | | 10-11-15-46 | | Colombia | | |
| 58 | 30 | 24 | | 18 | 27 | 16 | | 31 | 25 | 7 | | 16 | 8 | 6 | | 10-11-14-47 | | Colombia | | |
| 58 | 30 | 24 | | 18 | 26 | 16 | | 31 | 25 | 7 | | 16 | 8 | 6 | | 10-11-14-05 | | Nigeria | | |
| 58 | 30 | 24 | | 18 | 24 | 16 | | 31 | 25 | 7 | | 16 | 8 | 6 | | 10-11-03-69 | | Venezuela | | |
| 58 | 30 | 24 | | 18 | 24 | 16 | | 30 | 25 | 7 | | 16 | 8 | 6 | | 10-13-09-83 | | Nigeria | | |
| 58 | 30 | 24 | | 19 | 24 | 16 | | 31 | 25 | 7 | | 16 | 8 | 6 | | 10-21-06-36 | | Brazil | | |
| 58 | 30 | 24 | | 19 | 24 | 16 | | 31 | 25 | 7 | | 16 | 8 | 6 | | 10-21-06-29 | | Brazil | | |
| 58 | 30 | 24 | | 18 | 23 | 16 | | 31 | 25 | 7 | | 16 | 8 | 6 | | 10-08-01-06 | | Venezuela | | |
| 58 | 30 | 24 | | 18 | 27 | 16 | | 31 | 25 | 7 | | 16 | 8 | 6 | | 10-21-07-06 | | Brazil | | |
| 58 | 30 | 25 | | 18 | 27 | 16 | | 31 | 25 | 7 | | 16 | 8 | 6 | | 10-05-15-20 | | Venezual | | |
| 24 | 9 | 9 | | 20 | 11 | 14 | | 24 | 26 | 6 | | 16 | 11 | 6 | | 10-13-10-57 | | Iran | | V |
| 24 | 9 | 9 | | 20 | 11 | 14 | | 24 | 26 | 6 | | 16 | 11 | 6 | | 10-13-08-58 | | Iran | | |
| 24 | 9 | 9 | | 20 | 11 | 14 | | 24 | 26 | 6 | | 16 | 11 | 6 | | 10-13-10-56 | | Iran | | |
| 24 | 9 | 9 | | 20 | 11 | 14 | | 24 | 26 | 6 | | 16 | 11 | 6 | | 10-13-10-90 | | Iran | | |
| 24 | 9 | 8 | | 20 | 11 | 14 | | 24 | 26 | 6 | | 16 | 11 | 6 | | 10-11-10-18 | | Iran | | |
20
40
80
100
60
Similarity (%)
