# Supplementary material for: SCY-247, a novel second-generation triterpenoid antifungal, demonstrates high in vitro activity against genetically diverse Candida auris isolates, including FKS1 mutants
Source: J Antimicrob Chemother. 2025 Jul 17;80(11):3165–6. doi: 10.1093/jac/dkaf240 (PMC12598769; doi:10.1093/jac/dkaf240)
Supplement: dkaf240_Supplementary_Data [file dkaf240_supplementary_data.zip › Supplementary materials.docx]

**Appendix**

**Table S1: Isolate overview and MICs against common antifungals according to EUCAST E.Def v7.4 guidelines and *FKS1* mutations.** All MICs in mg/L.

| **ID** | **Clade** | **Country** | **FLU** | **VOR** | **POS** | **ITC** | **ISA** | **AMB** | **AFG** | **MFG** | **SCY-247** | ***FKS1*** |
| --- | --- | --- | --- | --- | --- | --- | --- | --- | --- | --- | --- | --- |
| 10-13-13-02 | I | Kuwait | ≥64 | 8 | 0.06 | 0.25 | 1 | 0.5 | 0.25 | 0.25 | 0.25 | S639Y |
| 10-13-13-07 | I | Kuwait | 64 | 0.25 | 0.016 | 0.016 | 0.016 | 0.5 | 4 | 8 | 0.5 | S639F |
| 10-13-13-08 | I | Kuwait | 64 | 0.25 | 0.016 | 0.016 | 0.016 | 1 | 8 | 8 | 0.5 | M690V |
| 10-13-13-09 | I | Kuwait | ≥64 | 1 | 0.125 | 0.125 | 0.5 | 0.5 | 2 | 1 | 1 | S639T |
| 10-13-14-35 | I | Kuwait | ≥64 | 1 | 0.06 | 0.06 | 0.25 | 0.25 | ≥8 | ≥8 | 4 | Δ635F |
| 10-13-14-39 | I | Kuwait | ≥64 | 1 | 0.06 | 0.06 | 0.25 | 1 | 0.03 | 0.06 | 0.125 | WT |
| 10-13-14-41 | I | Kuwait | ≥64 | 0.5 | 0.03 | 0.06 | 0.25 | 0.5 | ≥8 | ≥8 | 4 | Δ635F |
| 10-13-13-15 | I | Kuwait | ≥64 | 1 | 0.03 | 0.06 | 0.25 | 0.5 | 4 | 8 | 1 | S639Y |
| 10-13-09-84 | I | Brazil | 8 | 0.06 | 0.03 | 0.06 | 0.06 | 0.25 | 0.03 | 0.03 | 0.5 | WT |
| 10-14-04-57 | I | Greece | ≥64 | 4 | 0.03 | 0.03 | 0.5 | 0.25 | 0.06 | 0.06 | 0.25 | WT |
| 10-12-12-18 | I | India | ≥64 | 1 | 0.125 | 0.25 | 0.5 | 0.5 | 0.06 | 0.06 | 0.5 | WT |
| 10-13-15-89 | I | Kuwait | 64 | 0.125 | 0.008 | 0.016 | 0.06 | 1 | 8 | ≥8 | 0.5 | S639F |
| 10-14-04-56 | I | Greece | ≥64 | 4 | 0.06 | 0.03 | 1 | 1 | 4 | 8 | 2 | S639P |
| 10-13-14-50 | I | Brazil | 2 | 0.03 | 0.016 | 0.03 | 0.016 | 0.25 | 0.06 | 0.06 | 0.06 | WT |
| 10-13-15-86 | I | Kuwait | ≥64 | 0.25 | 0.008 | 0.016 | 0.03 | 0.5 | 8 | ≥8 | 1 | S639P |
| 10-05-12-41 | I | India | ≥64 | 2 | 0.06 | 0.03 | 0.5 | 0.25 | 0.06 | 0.016 | 0.25 | WT |
| 10-11-10-21 | I | Oman | ≥64 | 1 | 0.06 | 0.25 | 0.5 | 1 | 0.03 | 0.03 | 0.125 | WT |
| 10-08-13-13 | I | Kuwait | 32 | 0.125 | 0.008 | 0.008 | 0.008 | 0.5 | 4 | ≥8 | 0.5 | S639F |
| 10-08-13-15 | I | Kuwait | ≥64 | 1 | 0.016 | 0.016 | 0.5 | 0.5 | 8 | 8 | 0.5 | S639F |
| 10-13-03-68 | I | India | ≥64 | 2 | 0.25 | 0.125 | 0.5 | 0.5 | 0.03 | 0.03 | 0.25 | WT |
| 10-13-03-74 | I | India | ≥64 | 2 | 0.25 | 0.125 | 0.5 | 0.5 | 0.03 | 0.03 | 0.5 | WT |
| 10-13-05-26 | I | Kuwait | 32 | 0.5 | 0.016 | 0.016 | 0.03 | 0.5 | 0.06 | 0.125 | 0.25 | WT |
| 10-12-18-17 | I | Pakistan | ≥64 | 1 | 0.25 | 0.125 | 0.5 | 0.25 | 0.03 | 0.06 | 0.25 | WT |
| 10-13-09-86 | I | Brazil | ≥64 | 2 | 0.06 | 0.06 | 0.25 | 0.5 | 0.06 | 0.03 | 0.125 | WT |
| 10-11-10-22 | I | Oman | 8 | 0.06 | 0.03 | 0.06 | 0.06 | 0.5 | 0.03 | 0.03 | 0.06 | WT |
| 10-11-10-24 | I | Oman | 32 | 0.125 | 0.008 | 0.016 | 0.008 | 1 | 0.03 | 0.03 | 0.5 | WT |
| 10-11-13-09 | I | India | ≥64 | 2 | 0.125 | 0.125 | 0.5 | 0.25 | ≥8 | ≥8 | 1 | S639P |
| 10-11-13-17 | I | India | 32 | 0.125 | 0.008 | 0.016 | 0.008 | 1 | 8 | ≥8 | 0.25 | S639P |
| 10-05-12-42 | I | India | ≥64 | 2 | 0.016 | 0.06 | 0.25 | 0.25 | 0.03 | 0.03 | 0.25 | WT |
| 10-05-13-95 | I | India | ≥64 | 1 | 0.03 | 0.125 | 0.5 | 0.5 | 0.03 | 0.06 | 0.25 | WT |
| 10-05-13-96 | I | India | ≥64 | 4 | 0.06 | 0.25 | 0.5 | 0.5 | 0.03 | 0.03 | 0.25 | WT |
| 10-12-18-26 | I | Pakistan | ≥64 | 2 | 0.06 | 0.125 | 0.125 | 0.25 | 0.06 | 0.03 | 0.25 | WT |
| 10-12-16-85 | I | India | ≥64 | 0.5 | 0.06 | 0.03 | 0.03 | 0.5 | 0.06 | 0.03 | 0.125 | WT |
| 10-12-17-79 | I | India | ≥64 | 0.125 | 0.008 | 0.016 | 0.016 | 0.5 | 0.03 | 0.125 | 0.125 | WT |
| 10-12-17-85 | I | India | ≥64 | 0.5 | 0.06 | 0.125 | 0.03 | 0.5 | 0.03 | 0.03 | 0.125 | WT |
| 10-03-10-63 | II | South Korea | ≥64 | 1 | 0.25 | 0.25 | 1 | 0.5 | 0.03 | 0.016 | 0.06 | WT |
| 10-03-10-65 | II | Japan | 2 | 0.016 | 0.016 | 0.016 | 0.016 | 1 | 0.03 | 0.03 | 0.031 | WT |
| 10-03-10-62 | II | South Korea | ≥64 | 1 | 0.25 | 0.25 | 1 | 0.5 | 0.125 | 0.06 | 0.06 | WT |
| 10-05-15-50 | III | South Africa | ≥64 | 0.25 | 0.016 | 0.016 | 0.008 | 0.5 | 0.03 | 0.03 | 0.25 | WT |
| 10-05-15-52 | III | South Africa | 64 | 0.5 | 0.03 | 0.03 | 0.016 | 0.5 | 0.125 | 0.03 | 0.5 | WT |
| 10-05-15-54 | III | South Africa | 64 | 0.5 | 0.03 | 0.03 | 0.016 | 0.5 | 0.03 | 0.06 | 0.5 | WT |
| 10-05-15-49 | III | South Africa | ≥64 | 1 | 0.06 | 0.125 | 0.06 | 1 | 0.03 | 0.03 | 0.5 | WT |
| 10-08-12-01 | III | Spain | ≥64 | 1 | 0.06 | 0.06 | 0.06 | 0.5 | 0.06 | 0.06 | 0.125 | WT |
| 10-08-11-92 | III | Spain | ≥64 | 2 | 0.03 | 0.125 | 0.125 | 0.25 | 0.06 | 0.03 | 0.125 | WT |
| 10-08-11-93 | III | Spain | ≥64 | 0.5 | 0.06 | 0.016 | 0.03 | 0.5 | 0.06 | 0.125 | 0.125 | WT |
| 10-08-12-02 | III | Spain | ≥64 | 0.5 | 0.016 | 0.03 | 0.016 | 0.5 | 0.03 | 0.125 | 0.125 | WT |
| 10-08-12-04 | III | Spain | ≥64 | 0.25 | 0.016 | 0.016 | 0.016 | 0.5 | 0.06 | 0.03 | 0.125 | WT |
| 10-08-12-05 | III | Spain | ≥64 | 0.5 | 0.016 | 0.03 | 0.03 | 1 | 0.03 | 0.03 | 0.125 | WT |
| 10-13-09-82 | IV | Nigeria | 32 | 1 | 0.03 | 0.016 | 0.125 | 0.5 | 0.06 | 0.03 | 0.125 | WT |
| 10-13-09-83 | IV | Nigeria | ≥64 | 0.5 | 0.06 | 0.06 | 0.125 | 0.25 | 0.06 | 0.06 | 0.25 | WT |
| 10-21-06-35 | IV | Brazil | 2 | 0.016 | 0.008 | 0.016 | 0.016 | 0.5 | 0.016 | 0.03 | 0.125 | WT |
| 10-21-06-36 | IV | Brazil | 1 | 0.008 | 0.008 | 0.008 | 0.008 | 1 | 0.03 | 0.03 | 0.06 | WT |
| 10-21-06-29 | IV | Brazil | 2 | 0.03 | 0.008 | 0.016 | 0.016 | 0.5 | 0.03 | 0.016 | 0.125 | WT |
| 10-11-14-05 | IV | Colombia | 4 | 0.06 | 0.03 | 0.016 | 0.06 | 1 | 0.03 | 0.06 | 0.125 | WT |
| 10-11-14-46 | IV | Colombia | 4 | 0.03 | 0.03 | 0.016 | 0.06 | 0.25 | 0.03 | 0.06 | 0.25 | WT |
| 10-11-14-47 | IV | Colombia | 4 | 0.06 | 0.016 | 0.016 | 0.06 | 0.5 | 0.03 | 0.125 | 0.06 | WT |
| 10-08-01-06 | IV | Venezuela | ≥64 | 4 | 0.125 | 0.125 | 0.5 | 0.5 | 0.03 | 0.03 | 0.125 | WT |
| 10-11-03-69 | IV | Colombia | 4 | 0.06 | 0.03 | 0.06 | 0.06 | 0.25 | 0.06 | 0.06 | 0.06 | WT |
| 10-05-15-20 | IV | Venezuela | ≥64 | 4 | 0.03 | 0.125 | 0.5 | 0.5 | 0.06 | 0.125 | 0.125 | WT |
| 10-21-07-06 | IV | Brazil | 2 | 0.06 | 0.008 | 0.008 | 0.016 | 1 | 0.03 | 0.016 | 0.25 | WT |
| 10-13-08-58 | V | Iran | ≥64 | 8 | 0.06 | 0.25 | 0.5 | 1 | 0.03 | 0.06 | 0.125 | WT |
| 10-11-10-18 | V | Iran | 8 | 0.125 | 0.06 | 0.125 | 0.125 | 0.25 | 0.03 | 0.03 | 0.125 | WT |
| 10-13-10-57 | V | Iran | 2 | 0.016 | 0.016 | 0.016 | 0.008 | 1 | 0.03 | 0.06 | 0.25 | WT |
| 10-13-10-90 | V | Iran | ≥64 | 1 | 0.06 | 0.125 | 0.5 | 1 | 0.03 | 0.06 | 0.06 | WT |
| 10-13-10-56 | V | Iran | 2 | 0.016 | 0.008 | 0.016 | 0.008 | 0.5 | 0.125 | 0.03 | 0.125 | WT |

FLU, fluconazole; VOR, voriconazole; POS, posaconazole; ITC, itraconazole; ISA, isavuconazole; AMB, amphotericin B; AFG, anidulafungin; MFG, micafungin.
